# Kabuki Syndrome: Epigenetic Mechanisms, DNA Methylation Signatures, and Diagnostic Advances

**DOI:** 10.3390/ijms27219490

**Published:** 2026-10-24

**Authors:** Kseniya Zabudskaya, Oxana Ryzhkova

**Affiliations:** Research Centre for Medical Genetics, Moskvorechye St., 1, 115522 Moscow, Russia

**Keywords:** Kabuki syndrome, *KMT2D*, *KDM6A*, DNA methylation, episignature, epigenetic diagnostics

## Abstract

Kabuki syndrome (KS) is a rare multisystem developmental disorder characterized by distinctive facial features, developmental delay, hypotonia, and variable congenital anomalies. Most molecularly confirmed cases are associated with pathogenic variants in *KMT2D* (KS type 1) or *KDM6A* (KS type 2), genes encoding regulators of chromatin modification and gene expression. However, a subset of individuals with a clinical diagnosis of KS remains molecularly unresolved, while variants of uncertain significance may complicate diagnostic interpretation. DNA methylation episignatures have emerged as complementary molecular biomarkers that may support the interpretation of uncertain genetic findings and help clarify selected diagnostically unresolved cases. This review examines the molecular and epigenetic mechanisms underlying KS, summarizes the evidence for KS-associated DNA methylation episignatures, and discusses their potential applications in clinical diagnostics. Particular attention is given to the interpretation of episignature classifier results, the available evidence on diagnostic performance, and methodological limitations, including the representation of different variant classes and the interpretation of negative or inconclusive results. The review also considers genotype–phenotype correlations, genetic counseling, and clinical management in the context of current diagnostic approaches. Although episignature analysis may provide valuable functional evidence complementary to genetic testing, its results should be interpreted alongside clinical findings and molecular data. Further validation in well-characterized cohorts is needed to define its diagnostic utility across the full spectrum of KS-associated genetic variation.

## 1. Introduction

Kabuki syndrome (KS; OMIM #147920 and #300867) is a rare, genetically heterogeneous developmental disorder characterized by distinctive facial features, postnatal growth deficiency, developmental delay and/or intellectual disability, and variable congenital anomalies affecting multiple organ systems. First described independently by Niikawa et al. and Kuroki et al. in 1981, KS was named after the resemblance of the characteristic facial appearance to the makeup of performers in traditional Japanese Kabuki theatre [1,2]. The clinical spectrum includes cardiac and renal malformations, skeletal and dental abnormalities, hypotonia, feeding difficulties, hearing impairment, and immune and endocrine dysfunction [3,4,5]. The expression of individual features varies with age and between affected individuals, which may complicate clinical recognition.

Pathogenic variants in *KMT2D* and *KDM6A* account for the two principal molecular forms of KS, designated KS type 1 and KS type 2, respectively [6]. *KMT2D* encodes a histone methyltransferase involved in the regulation of histone H3 lysine 4 methylation, whereas *KDM6A* encodes a histone demethylase that removes repressive H3K27 methylation marks. Both proteins participate in chromatin regulation and developmental gene expression. Their distinct but interconnected functions provide a mechanistic basis for investigating epigenetic changes associated with KS.

Despite advances in molecular genetic testing, diagnostic uncertainty persists in some individuals with a clinical presentation suggestive of KS. Potential challenges include variants of uncertain significance (VUS), mosaic variants, structural or non-coding variants that may not be reliably detected by every testing strategy, and cases in which no causative variant is identified. The proportion of unresolved cases depends on the population investigated, the clinical criteria used, and the methods and extent of genetic testing. Accordingly, the absence of an identified pathogenic variant should be distinguished from the absence of a clinical diagnosis, and estimates of the molecular diagnostic yield should be interpreted in the context of the underlying study population.

DNA methylation analysis offers a complementary approach because disturbances in chromatin regulators can be associated with reproducible changes in DNA methylation patterns. Disease-associated patterns, commonly referred to as episignatures, can be assessed using molecular classification approaches and may provide functional evidence to support the interpretation of selected genetic variants. In KS, this approach is particularly relevant to the interpretation of VUS and the investigation of selected unresolved cases. However, an episignature result is not equivalent to a clinical diagnosis or a direct demonstration that an individual variant is pathogenic; its interpretation depends on the assay, reference data, classification criteria, and clinical and molecular context [7].

The aim of this review is to summarize the molecular and epigenetic mechanisms underlying KS, critically evaluate the available evidence on KS-associated DNA methylation episignatures, and discuss their potential diagnostic applications and limitations. We focus on the relationship between *KMT2D*/*KDM6A* dysfunction and DNA methylation changes, the interpretation of episignature-based results in patients with VUS or unresolved molecular findings, and the remaining challenges in translating these methods into clinical practice. Genotype–phenotype correlations, genetic counseling, and clinical management are also considered where they inform the interpretation and application of current diagnostic approaches.

To ensure a comprehensive and objective overview of the topic, a non-systematic literature search was conducted across major electronic databases, including PubMed, Google Scholar, and Scopus. The search covered literature published using keywords and their combinations, such as [‘Kabuki’, ‘*KMT2D*’, ‘*KDM6A*’, ‘DNA methylation’, ‘episignature’]. Articles were selected for inclusion based on their relevance to the review’s core themes, methodological rigor, and contribution to the current understanding of the field. Only peer-reviewed articles published in English were considered.

## 2. Diagnostic Criteria

The clinical diagnosis of KS has evolved since its initial description by Niikawa et al. and Kuroki et al. in 1981 [1,2]. In 1988, Niikawa et al. summarized five characteristic features: distinctive facial dysmorphism, skeletal anomalies, persistent fetal fingertip pads, mild-to-moderate intellectual disability, and postnatal growth deficiency [5]. The characteristic facial features include long palpebral fissures with eversion of the lateral third of the lower eyelid, arched eyebrows with thinning or notching of the lateral third, a short columella with a depressed nasal tip, and large, prominent, or cupped ears. Although these findings remain important for clinical recognition, the phenotypic spectrum of KS is broad and varies among affected individuals and across different ages.

The identification of pathogenic variants in *KMT2D* in 2010 and *KDM6A* in 2012 established the molecular basis of KS types 1 and 2, respectively [8,9,10]. These discoveries provided a basis for integrating clinical findings with molecular genetic testing. Subsequently, Makrythanasis et al. proposed a phenotypic scoring system intended to predict the likelihood of *KMT2D*-associated KS in clinically suspected cases [11]. In a cohort of 86 individuals, the mean score was higher among patients with an identified pathogenic *KMT2D* variant than among those without an identified clinically significant variant in *KMT2D* (6.1 versus 4.5) [11]. However, this scoring system has not been adopted as a formal diagnostic standard [12]. Because its principal purpose was to assess the likelihood of a *KMT2D*-associated diagnosis, it should be distinguished from the international consensus criteria used in current clinical assessment.

The clinical presentation of KS may include congenital heart and renal anomalies, skeletal and dental abnormalities, hypotonia, developmental delay, feeding difficulties, hearing impairment, and immune, visual, gastrointestinal, and endocrine abnormalities [4,5,13]. The frequency and recognition of individual features may vary with age and between reported populations. Apparent differences between ethnic groups should be interpreted cautiously because they may reflect differences in ascertainment, sample size, access to diagnostic services, and population representation, rather than established biological differences [11,12,14].

In 2019, international consensus diagnostic criteria were proposed to distinguish definite, probable, and possible KS [14]. These criteria provide a standardized framework for clinical evaluation while recognizing that the phenotype may be age-dependent and that molecular testing may not always establish a diagnosis. They are particularly relevant when genetic testing is unavailable or inconclusive, or when a patient carries a variant of uncertain significance (VUS) in *KMT2D* or *KDM6A*.

Under the consensus framework, a definite diagnosis can be established in an individual of any age who has infantile hypotonia and developmental delay, together with either the characteristic facial phenotype or an appropriate pathogenic or likely pathogenic variant in *KMT2D* or *KDM6A*. The characteristic facial phenotype is defined by long palpebral fissures with eversion of the lateral third of the lower eyelid and at least two of the following features: arched and broad eyebrows with a notch or thinning of the lateral third; a short columella with a depressed nasal tip; large, prominent, or cupped ears; and persistent prominent fetal fingertip pads [14]. A molecular diagnosis requires a heterozygous pathogenic or likely pathogenic variant in *KMT2D* or a heterozygous or hemizygous pathogenic or likely pathogenic variant in *KDM6A*, interpreted in accordance with the relevant inheritance pattern and clinical context.

A probable diagnosis is considered in individuals with infantile hypotonia, developmental delay and/or intellectual disability, long palpebral fissures with eversion of the lateral third of the lower eyelid, and at least three additional characteristic clinical features. A possible diagnosis may be considered in individuals with developmental delay and/or intellectual disability, at least two additional clinical features, and at least one of the characteristic dysmorphic findings specified in the consensus criteria [14]. Additional findings supporting the clinical assessment are summarized in Table 1.

For individuals with a probable or possible clinical diagnosis, molecular genetic testing can help establish the underlying cause and refine diagnostic interpretation. Testing strategies may include targeted analysis of *KMT2D* and *KDM6A*, gene panels, chromosomal microarray analysis, whole-exome sequencing, or whole-genome sequencing, depending on the clinical presentation and the testing already performed. The diagnostic yield depends on the methods used and their ability to detect different variant classes, including sequence variants, copy-number changes, structural variants, and mosaic variants.

Importantly, clinical diagnosis, molecular diagnosis, and variant classification are related but distinct outcomes. A patient may meet clinical criteria for KS without an identifiable causative variant, whereas the detection of a VUS does not by itself establish a molecular diagnosis. In selected cases, additional evidence from segregation analysis, functional studies, or DNA methylation episignature testing may help clarify the interpretation of genetic findings. Episignature analysis is a complementary approach rather than a substitute for clinical assessment or appropriate molecular testing; its role and limitations are discussed in the following section.

International consensus criteria provide a standardized framework for the clinical evaluation of KS. However, their application may be influenced by age-dependent phenotypic features, clinical ascertainment, and access to molecular genetic testing. Clinical criteria and molecular testing should therefore be considered complementary components of the diagnostic process.

## 3. Molecular Mechanism

KS is a complex congenital disorder primarily caused by mutations in the genes *KMT2D*, encoding a histone H3 lysine 4 (H3K4) methyltransferase, and *KDM6A*, encoding a histone H3 lysine 27 (H3K27) demethylase. Both enzymes play critical roles in epigenetic regulation, controlling chromatin architecture and gene expression during development. Disruption of their function leads to widespread changes in transcriptional programs essential for neural crest cell function and other developmental pathways implicated in KS [17,18].

Histone modification and chromatin regulation: *KMT2D* catalyzes mono- and tri-methylation of H3K4 (H3K4me1 and H3K4me3), epigenetic marks characteristic of active enhancers and promoters, respectively. Loss-of-function mutations in *KMT2D* impair this methyltransferase activity, leading to altered enhancer landscapes and transcriptional dysregulation. Importantly, studies in peripheral blood mononuclear cells (PBMCs) of KS patients revealed reduced enhancer signals at immune-related gene loci, correlating with immune deficiency phenotypes frequent in KS, as well as increased transcription of metabolic genes, illustrating the pleiotropic effects of *KMT2D* haploinsufficiency [19].

Additionally, *KMT2D* mutations result in novel de novo enhancer formation in genes implicated in diverse cellular functions, further complicating the transcriptional output [20].

*KDM6A* demethylates the repressive H3K27me3 mark, promoting chromatin accessibility and gene activation. Mouse studies indicate that neural crest (NC) deletion of *KDM6A* (also known as UTX) models KS craniofacial, cardiac, and growth defects, supporting the classification of KS as a neurocristopathy. Intriguingly, demethylase-independent functions of *KDM6A* also contribute to neural crest gene regulation, as many target genes do not exhibit altered H3K27me3 or H3K4me3 marks in mutants, suggesting additional roles for *KDM6A* as a chromatin scaffold or recruiter of transcriptional machinery [17,18].

***Neural crest cell dysfunction:*** Neural crest cells (NCCs) are pluripotent progenitors essential for the craniofacial skeleton, heart, and other tissues affected in KS. *KMT2D* haploinsufficiency impairs NCC differentiation, migration, and gene regulation, disturbing key developmental pathways, including expression of transcription factors like TFAP2A, a master regulator of NCC lineage progression. Mouse and Xenopus models demonstrate disrupted NCC formation and migration, contributing to developmental craniofacial anomalies such as cleft palate, mandible hypoplasia, and defective cranial base ossification seen in KS patients [21,22,23].

***Brain development and anomalies:*** While intellectual disability is common in KS, structural brain anomalies are less frequently described. However, recent reports of holoprosencephaly associated with *KMT2D* mutations expand the neurological phenotype spectrum and underscore the importance of precise temporal and spatial epigenetic regulation for early brain development [24].

Further, *KMT2D* deficiency leads to premature neuronal differentiation and disruption of oxygen-responsive gene pathways in neural stem/progenitor cells, implicating metabolic and hypoxia response defects in KS neuropathology [25].

***Epigenetic diagnostics and mosaicism:*** Genome-wide DNA methylation episignatures specific to KS assist in diagnosis, especially when genetic testing is inconclusive. These methylation signatures reflect downstream epigenetic consequences of *KMT2D*/*KDM6A* variants and can detect mosaic cases with low-level variant allele fractions, thereby expanding diagnostic sensitivity [26,27].

***Therapeutic insights:*** Experimental interventions targeting epigenetic dysregulation offer promise. Supplementation with the endogenous ketone metabolite beta-hydroxybutyrate (βHB), a histone deacetylase inhibitor, modulates histone acetylation and methylation, showing potential to restore chromatin states and ameliorate deficits in KS mouse models [28]. This epigenetic modifier approach could open avenues for novel personalized therapies.

***Non-canonical functions:*** Beyond their catalytic roles, *KMT2D* and *KDM6A* serve as recruitment platforms for alternative chromatin remodeling complexes and transcriptional regulators, mediating genome organization and transcriptional state establishment independent of enzymatic activity. This multifunctionality likely underpins phenotypic variability and complex molecular pathology observed in KS [17].

Together, these molecular insights emphasize disrupted chromatin modification and neural crest dysfunction as central drivers of the multisystemic phenotype in Kabuki syndrome, integrating epigenetic, developmental, and neurological perspectives into a cohesive pathophysiological framework.

Pathogenic variants in *KMT2D* and *KDM6A* associated with KS include loss-of-function changes, such as nonsense, frameshift, and canonical splice-site variants, which can lead to haploinsufficiency, as well as missense variants [4]. The functional consequences of missense variants may depend on their location and effects on protein structure or interactions. In *KMT2D*, some missense variants have been proposed to impair histone methyltransferase activity by disrupting interactions with the WRAD complex or by altering protein secondary structure [29,30,31,32]. These mechanisms should be interpreted in the context of the specific variants and experimental evidence available.

Additional variant classes have been reported as “disease-causing mutations” in the HGMD Professional database (version 2025.1) for both genes, including deep intronic variants, copy-number changes, complex genomic rearrangements, and translocations (accessed on 11 October 2026).

Although most cases are sporadic, inherited variants and somatic or gonadal mosaicism have also been reported [33,34,35,36]. These findings are relevant to molecular diagnosis and genetic counseling because variant detection and recurrence-risk assessment may depend on the type of variant, the tissues tested, and the sensitivity of the analytical method.

The phenotypic spectrum associated with *KMT2D* variants extends beyond classic KS. Missense variants affecting a highly conserved region encoded by exons 38 and 39 have been associated with a distinct disorder characterized by choanal atresia, athelia, thyroid abnormalities, differences in sexual development, and short stature (MIM 620186) [37,38]. In addition, *KMT2D* variants have been reported in individuals with isolated alobar holoprosencephaly [39]. These observations highlight the phenotypic variability associated with *KMT2D* and the importance of interpreting genetic findings in conjunction with clinical presentation and variant-specific evidence.

## 4. Episignatures

The molecular diagnosis of KS primarily relies on next-generation sequencing (NGS), including targeted gene panels and whole-exome sequencing, while whole-genome sequencing may be considered in selected cases. Although these methods enable the identification of pathogenic variants in *KMT2D* and *KDM6A*, diagnostic uncertainty may persist because of variants of uncertain significance (VUS), variants not detected by the assay used, or negative results despite a suggestive clinical phenotype. The detection of copy-number, deep intronic, and low-level mosaic variants depends on the sequencing platform, coverage, and bioinformatic pipeline. Segregation studies, functional assays, and reanalysis of sequencing data may help resolve uncertain findings but are not always conclusive.

Because *KMT2D* and *KDM6A* regulate chromatin and gene expression, pathogenic variants in these genes may produce downstream alterations in DNA methylation. Genome-wide DNA methylation analysis can identify reproducible disease-associated patterns, termed episignatures, which may serve as biomarkers of disorders involving epigenetic regulation [40,41,42]. DNA methylation is commonly assessed at CpG sites, and differentially methylated positions (DMPs) or regions are identified by comparing affected individuals with appropriate controls. The resulting methylation profiles can be used to develop classifiers that assess whether an individual’s profile matches a reference episignature.

The first reported DNA methylation signature associated with KS1, caused by pathogenic variants in KMT2D, comprised 221 differentially methylated CpG sites [43]. However, the number of discriminatory sites alone does not establish clinical diagnostic performance. Interpretation requires consideration of the discovery cohort, control selection, methylation platform, data preprocessing, classifier development, and independent validation. Subsequent studies have also highlighted the influence of normalization procedures, missing probes, and differences between methylation platforms on classification performance [44].

Clinical episignature analysis may complement sequencing in two principal settings: supporting the interpretation of a VUS in a relevant disease-associated gene and evaluating individuals with a suggestive phenotype but inconclusive molecular findings. These applications should be distinguished because a positive episignature may support the functional interpretation of a variant without independently establishing every aspect of the clinical diagnosis.

The EpiSign classification framework uses a supervised machine learning classifier to compare an individual’s methylation profile with reference disease-associated patterns. The methylation variant pathogenicity (MVP) score ranges from 0 to 1 and reflects the classifier’s confidence that the profile matches a particular episignature; it is not a pathogenicity score for an individual DNA methylation variant. In the published EpiSign framework, an MVP score above 0.5 has generally been used as one component of a positive classification, together with concordant hierarchical clustering and multidimensional scaling results [45]. This threshold should be interpreted within the context of the relevant classifier and laboratory workflow rather than as a universal diagnostic criterion.

The diagnostic performance of DNA methylation episignatures in Kabuki syndrome (KS) depends on the classifier used and the validation strategy. Earlier studies established the potential utility of genome-wide DNA methylation profiling in supporting the diagnosis of KS, resolving uncertain molecular findings, and guiding further genomic investigations [26,27]. More recently, a next-generation KS1 episignature was developed and evaluated in independent validation cohorts, achieving a reported sensitivity of 97% and specificity of 100% [46]. The classifier also demonstrated potential utility in interpreting *KMT2D* variants and assessing mosaic cases. However, these performance estimates apply to the specific classifier and study cohorts and should not be generalized to all episignature assays. Further evaluation in larger and more diverse clinical cohorts is needed to establish its performance across different variant classes and clinical presentations.

Important limitations remain. A negative result does not necessarily exclude KS, particularly when the patient’s variant class, mosaic fraction, biological sample, or methylation profile is not adequately represented in the reference data. The extent to which episignatures distinguish KS1 from KS2 and reliably detect all molecular classes requires consideration of the specific assay and supporting evidence. Accordingly, DNA methylation analysis should be interpreted alongside clinical findings, sequencing results, variant classification, and, when appropriate, family segregation data.

## 5. Genetic Counseling

KS type 1, caused by a variant in the *KMT2D* gene, has an autosomal dominant pattern of inheritance. Most often, the variant in patients arises *de novo* (that is, it is absent in the parents), and in that case the recurrence risk in the family approaches the general population risk (taking into account the possibility of gonadal mosaicism, the risk of having a child with KS is estimated at about 1%) [47,48].

In the rare case when the causative variant in the *KMT2D* gene is inherited from one of the parents, the risk for siblings is 50%.

KS type 2, caused by a variant in the *KDM6A* gene, has an X-linked dominant pattern of inheritance. If the mother has a clinically significant variant in the *KDM6A* gene, the probability of transmitting it in each pregnancy is 50%.

When planning a pregnancy, it is recommended to seek consultation with a geneticist to obtain complete information.

## 6. Management of Patients

The management of KS requires an individualized, multidisciplinary approach because of its variable multisystem manifestations. Although the diagnosis can be established in infancy, characteristic facial features may become more apparent with age, and additional manifestations may emerge during childhood or adolescence [14,49].

Prenatal findings are variable and nonspecific, including increased nuchal translucency, cardiac or renal anomalies, fetal growth restriction, and abnormalities of amniotic fluid volume [50,51]. When KS is suspected prenatally, genetic counseling and appropriate molecular testing should be considered.

### 6.1. Genotype–Phenotype Correlations

KS type 1 (KS1), associated with *KMT2D*, and KS type 2 (KS2), associated with *KDM6A*, share many clinical features but differ in the relative frequency of some manifestations. KS1 is more frequently associated with characteristic facial features, feeding difficulties, palatal and renal abnormalities, joint dislocations, and congenital heart defects. KS2 may present with less distinctive facial features and has been associated with hyperinsulinemic hypoglycemia, short stature, and dental abnormalities [32,49,52,53]. These associations are variable and do not reliably predict disease severity in an individual patient.

### 6.2. Clinical Assessment and Surveillance

Initial assessment should identify congenital abnormalities, developmental needs, and complications requiring treatment or follow-up. Subsequent surveillance should be individualized according to clinical findings. Table 2 illustrates a provisional patient management approach that is highly customizable, as definitive, rigid guidelines are currently lacking.

**Table 2:** Principal management domains in Kabuki syndrome.

Domain	Key Considerations
Cardiac and renal	Baseline assessment for congenital abnormalities and specialist follow-up when indicated
Growth and endocrine	Monitor growth and development; investigate hypoglycemia and endocrine abnormalities when clinically indicated
Feeding and gastrointestinal	Assess swallowing, feeding difficulties, growth, reflux, and bowel function
Immunological	Evaluate immune function and investigate recurrent infections or suspected autoimmune complications
Hearing and vision	Perform baseline assessments and arrange follow-up according to findings
Neurological and developmental	Monitor motor, cognitive, speech, and behavioral development; provide appropriate therapeutic and educational support
Musculoskeletal and dental	Assess joint laxity, hip abnormalities, scoliosis, dental anomalies, and palatal dysfunction
Surgery and psychosocial care	Individualize anesthetic planning and provide family support, genetic counseling, and educational resources

**Note:** This table summarizes the principal areas of care based on Management of Kabuki Syndrome: A Clinical Guideline (Version 1), developed by the Kabuki Syndrome Guideline Development Group [“https://www.orpha.net/pdfs/data/patho/Pro/en/KabukiGuidelines2011.pdf (accessed on 15 September 2026)”]. It is intended as a concise clinical reference and does not replace individualized assessment or current clinical guidance. The evidence supporting specific surveillance recommendations varies, and many recommendations are based primarily on expert consensus.

Although clinical management recommendations for Kabuki syndrome have been published, comprehensive guidelines supported by systematically evaluated evidence remain limited. Further research is needed to strengthen the evidence base and inform updated recommendations addressing the multisystem needs of affected individuals throughout their lives.

### 6.3. Long-Term Care and Evidence Limitations

Developmental interventions, including physiotherapy, occupational therapy, speech and language therapy, and individualized educational support, should be offered according to patient needs. Genetic counseling should address inheritance, recurrence risk, and implications for relatives. Transition planning is important to ensure continuity of care into adulthood.

There is currently no established disease-modifying treatment for KS; management remains primarily supportive and directed toward preventing and treating complications. Evidence for many surveillance recommendations derives from observational studies and expert consensus. Complementary interventions, such as music therapy or hippotherapy, should not be presented as established KS treatments because evidence of their specific efficacy remains limited. Similarly, therapeutic strategies targeting epigenetic dysregulation remain investigational, and findings from experimental models cannot be assumed to demonstrate clinical benefit in humans.

Overall, care should integrate clinical assessment, molecular diagnosis, genetic counseling, and multidisciplinary follow-up. Further studies are needed to strengthen the evidence base for long-term surveillance and treatment.

## 7. Conclusions

KS is a genetically heterogeneous developmental disorder associated primarily with pathogenic variants in *KMT2D* and *KDM6A*, which encode key regulators of chromatin modification and gene expression. Disruption of these epigenetic regulators affects developmental transcriptional programs and contributes to the characteristic multisystem phenotype. Although clinical diagnostic criteria and molecular genetic testing have substantially improved the recognition of KS, diagnostic uncertainty persists in individuals with suggestive clinical features but inconclusive genetic findings. The proportion of molecularly unresolved cases should be interpreted in the context of the diagnostic criteria applied, the genes and variant classes investigated, and the technologies used.

Advances in understanding the molecular mechanisms of KS have provided a rationale for investigating disease-associated DNA methylation episignatures as complementary diagnostic biomarkers. By detecting downstream epigenetic consequences of pathogenic variants, episignature analysis may provide additional functional evidence for the interpretation of selected variants of uncertain significance (VUS) and help resolve some cases in which conventional genetic testing does not establish a molecular diagnosis. Recent developments in episignature classification highlight the potential of this approach to improve diagnostic interpretation. Nevertheless, reported diagnostic performance varies according to the classifier, study population, validation strategy, and clinical context. Results from individual studies should therefore not be generalized to all methylation assays or patient groups.

The clinical interpretation of episignature testing requires consideration of several factors, including variant class, mosaicism, the representation of different molecular subtypes in reference cohorts, and the possibility of negative or inconclusive results. A positive episignature may support the interpretation of a candidate variant but should be assessed alongside the patient’s phenotype, sequencing data, segregation findings, and other available evidence. Conversely, a negative result does not necessarily exclude KS. Episignature analysis should therefore complement, rather than replace, clinical assessment and molecular genetic testing. Its contribution to resolving genetically unexplained cases should also be distinguished from its role in supporting the classification of VUS.

Further studies involving well-characterized and independently validated cohorts are needed to establish the sensitivity, specificity, and reproducibility of KS-associated episignatures across KS1 and KS2, different variant classes, and mosaic presentations. Comparative studies should also clarify the incremental diagnostic value of methylation profiling relative to sequencing, copy-number analysis, family segregation studies, functional assays, and reanalysis of genomic data. Standardization of laboratory procedures, classifier thresholds, and result interpretation will be important for ensuring reproducible findings and appropriate integration into clinical diagnostic pathways.

Beyond diagnosis, a better understanding of the relationship between chromatin dysregulation, DNA methylation patterns, and clinical variability may provide new insights into KS pathogenesis. However, proposed therapeutic approaches targeting epigenetic mechanisms remain investigational, and findings from experimental models should not be interpreted as evidence of established clinical efficacy. Future research should prioritize the validation of diagnostic biomarkers, the clarification of genotype–phenotype relationships, and the generation of robust clinical evidence to guide patient care.

Overall, integrating clinical evaluation, molecular genetic testing, and validated DNA methylation episignature analysis offers a promising framework for improving the diagnostic assessment of individuals with suspected KS. The principal challenge is to define the clinical contexts in which episignature testing provides reliable additional information, while clearly communicating its limitations. This approach may help reduce diagnostic uncertainty and support more accurate genetic counseling and individualized clinical management.

## Figures and Tables

**Table 1:** Additional features for the diagnosis of KS [14].

Group	Clinical Feature
Constitutional	Short stature ^1^
Craniofacial	Microcephaly ^2^ Cleft palate Lip pits Oligodontia and/or abnormal incisors ^3^ Progressive hearing loss
Cardiovascular	Congenital heart defects, except patent ductus arteriosus ^4^
Gastrointestinal	Feeding difficulties ^5^
Genitourinary	Hypospadias Renal anomalies
Skeletal and muscular	Non-traumatic joint dislocations, including congenital hip dislocation Brachydactyly
Endocrinological	Hyperinsulinemic hypoglycemia ^6^
Immunological	Hypogammaglobulinemia or low serum IgA Idiopathic thrombocytopenic purpura

^1^—Height 2 SD or more below the mean for age and sex. ^2^—Head circumference 2 SD or more below the mean for age and sex. ^3^—Absent upper lateral incisors, absent lower incisors, absent second premolars, ectopic upper molars, and/or shield-shaped upper incisors. ^4^—Approximately 70% of individuals with a causative variant in the *KMT2D* gene have congenital heart disease, in particular left-sided cardiac lesions and mitral valve anomalies. ^5^—Poorly coordinated sucking and swallowing, nasogastric tube placement, gastrostomy. ^6^—Premature thelarche [15], isolated growth hormone deficiency (GHD) [15], and combined pituitary hormone deficiency [16] have also been described in patients with KS.

## Data Availability

No new data were created or analyzed in this study. Data sharing is not applicable to this article.
